# Characterization of LEDGF/p75 Genetic Variants and Association with HIV-1 Disease Progression

**DOI:** 10.1371/journal.pone.0050204

**Published:** 2012-11-30

**Authors:** Peter Messiaen, Ward De Spiegelaere, Jose Alcami, Karen Vervisch, Petra Van Acker, Bruno Verhasselt, Pieter Meuwissen, Esther Calonge, Nuria Gonzalez, Felix Gutierrez-Rodero, Carmen Rodriguez-Martín, Erica Sermijn, Bruce Poppe, Dirk Vogelaers, Chris Verhofstede, Linos Vandekerckhove

**Affiliations:** 1 HIV Translational Research Unit, Department of General Internal Medicine and Infectious Diseases, Ghent University Hospital, Ghent, Belgium; 2 Unidad de Inmunopatología del SIDA, Centro Nacional de Microbiología, Instituto de Salud Carlos III, Madrid, Spain; 3 Center for Medical Genetics, Ghent University, Ghent, Belgium; 4 Department of Clinical Chemistry, Microbiology and Immunology, Ghent University, Ghent, Belgium; 5 Hospital General d'Elx, Alicante, Spain; 6 Centro Sanitario Sandoval, Madrid, Spain; Massachusetts General Hospital, United States of America

## Abstract

**Background:**

As Lens epithelium-derived growth factor (LEDGF/p75) is an important co-factor involved in HIV-1 integration, the LEDGF/p75-IN interaction is a promising target for the new class of allosteric HIV integrase inhibitors (LEDGINs). Few data are available on the genetic variability of LEDGF/p75 and the influence on HIV disease *in vivo*. This study evaluated the relation between LEDGF/p75 genetic variation, mRNA expression and HIV-1 disease progression in order to guide future clinical use of LEDGINs.

**Methods:**

Samples were derived from a therapy-naïve cohort at Ghent University Hospital and a Spanish long-term-non-progressor cohort. High-resolution melting curve analysis and Sanger sequencing were used to identify all single nucleotide polymorphisms (SNPs) in the coding region, flanking intronic regions and full 3′UTR of LEDGF/p75. In addition, two intronic tagSNPs were screened based on previous indication of influencing HIV disease. LEDGF/p75 mRNA was quantified in patient peripheral blood mononuclear cells (PBMC) using RT-qPCR.

**Results:**

325 samples were investigated from patients of Caucasian (n = 291) and African (n = 34) origin, including Elite (n = 49) and Viremic controllers (n = 62). 21 SNPs were identified, comprising five in the coding region and 16 in the non-coding regions and 3′UTR. The variants in the coding region were infrequent and had no major impact on protein structure according to SIFT and PolyPhen score. One intronic SNP (rs2737828) was significantly under-represented in Caucasian patients (P<0.0001) compared to healthy controls (HapMap). Two SNPs showed a non-significant trend towards association with slower disease progression but not with LEDGF/p75 expression. The observed variation in LEDGF/p75 expression was not correlated with disease progression.

**Conclusions:**

LEDGF/p75 is a highly conserved protein. Two non-coding polymorphisms were identified indicating a correlation with disease outcome, but further research is needed to clarify phenotypic impact. The conserved coding region and the observed variation in LEDGF/p75 expression are important characteristics for clinical use of LEDGINs.

## Background

Acquired Immunodeficiency Syndrome (AIDS) caused by the human immunodeficiency virus (HIV) is one of the major infectious diseases with 34 million people affected world-wide [1]. The introduction of combination antiretroviral treatment converted HIV/AIDS from a deadly disease into a chronic infection [2]. However, short- and long-term side-effects often challenge life-long antiviral treatment, prompting the need for new therapeutic strategies. This search has mainly been focussed on targeting viral enzymes as reverse transcriptase, protease and more recently integrase [3]. Different steps of the HIV-1 cycle are tightly dependent on cellular factors and accordingly host factors have become potential targets for HIV drug development. As an example, a first drug targeting the CCR5 co-receptor was approved for treatment of HIV-1 infection [4]. Further unravelling the host – virus interaction not only leads to better understanding of viral dynamics and disease progression, but also paves the way to validate new targets in the treatment of HIV-1 [5]. An increasing number of co-factors is being studied in the course of new strategies against HIV-1 infection [6].

An important and limiting step during cell infection is the integration of proviral DNA into host cell DNA, catalyzed by the viral enzyme integrase (IN). This requires the interaction with Lens epithelium-derived growth factor p75 (LEDGF/p75). By interacting with the catalytic core domain of IN, LEDGF/p75 functions as a chromatin tethering factor for the pre-integration complex, and targets HIV-1 integration towards actively transcribed genomic regions [7], [8], [9], [10], [11], [12], [13], [14]. LEDGF/p75 is a member of the hepatoma-derived growth factor (HDGF) related protein family (HRP-family) known as transcriptional co-activators for heat-shock and stress-related genes [15], [16], [17], [18], [19], [20]. It plays a role as anti-apoptotic protein in diverse oncologic settings [21], [22]. The essential role in HIV-1 replication has been elucidated by mutagenesis, RNA interference, transdominant expression of protein domains and knockout experiments [23], [24], [25], [26], [27]. Recently, allosteric inhibitors of the LEDGF/p75 - IN interaction (LEDGIN) were developed as a new class of antiretroviral treatment [28]. *In vitro* data show that LEDGINs also block the interaction between IN and HRP2, a second host protein of the HRP-family that contains a structurally similar integrase binding domain (IBD) and can substitute for HIV integration in cell lines with LEDGF/p75 knock-down [29].

Several Genome-wide association studies explored the influence of human genetic variation on HIV-1 replication and disease progression [30], [31], [32], [33], [34]. Genetic variants in genes associated with HLA-B*57:01 and the HLA-C gene region, together with the CCR5Δ32 variant can explain up to 13% of the observed variability in HIV-1 viremia [32] raising the need for further genetic studies to improve individualized prognosis in HIV-infected patients. Gene polymorphisms can be particularly important to predict response to treatment when drug targets are cellular host proteins [35]. Two studies investigated genetic variation in the LEDGF/p75 gene (known as PC4 and SFRS-1 interacting protein-1 or *PSIP1*). One study detected rare single nucleotide polymorphisms (SNPs) in the adjacent domains of LEDGF/p75 integrase binding domains (IBD) of Caucasian long-term non-progressor patients [36]. The other study genotyped five pre-defined tagSNPs in two South-African cohorts. They showed that the minor allele of one tagSNP was associated with higher CD4 T-cell count, lower viremia and reduced LEDGF/p75 mRNA expression during early infection and slower CD4 decline during the chronic phase. Another tagSNP minor allele was more prevalent in sero-positives and trended towards association with high likelihood of HIV-1 acquisition [37]. This data indicate there can be a correlation between genetic variants in the LEDGF/p75 domain and HIV disease outcome. However, a comprehensive analysis of all genetic variation in the coding region of LEDGF/p75 has not been performed so far and may provide information of additional variants with an influence on HIV disease progression.

The current study aimed at a comprehensive *in vivo* characterization of LEDGF/p75 on both genetic and mRNA level in a large and ethnically mixed HIV-1 infected patient cohort. We focused on the association of genetic variation in the full coding region+3′UTR of the LEDGF/p75 gene and HIV-1 disease progression and on the link of genetic variants with LEDGF/p75 and HRP2 mRNA expression levels.

## Methods

### Patient population

The study included chronically HIV-1 infected patients from the Aids Reference Center at Ghent University Hospital (n = 187) and HIV-1 long-term non-progressors (LTNP; n = 138) from the LTNP cohort of the Spanish AIDS Research Network (See Table 1). Samples from the LTNP cohort were kindly provided by the HIV BioBank integrated in the Spanish AIDS Research Network (RIS) [38]. Samples were processed and frozen immediately after collection. The Ghent patients had a therapy-naive follow-up period of at least two years with regular CD4 count and plasma HIV RNA determination (three times/year). They comprised patients from Caucasian (81.2%) as well as African (18.2%) descent. Data on HIV-1 subtype were available for 83% of patients with 70% of them harboring an HIV-1 B-subtype. The LTNP cohort were all of Caucasian origin, had a documented HIV-1 infection >10 years, consistent CD4 count above 500 cells/µl and viral load below 10,000 copies/ml in the absence of therapy. The slope of CD4 decline and the average plasma HIV viral load were determined for all patients based on at least four CD4+ T-cell counts and plasma HIV RNA measurements on samples collected with minimum three months' time interval during the therapy-naive follow-up period. Classification of patients according to disease progression was based on broadly applied clinical definitions [39], [40], [41], [42], [43]: LTNP elite controllers (LTNP-EC) are long-term non-progressors (10 year follow-up, CD4 >500cells/µL) maintaining an undetectable viral load without antiretroviral therapy (n = 48), LTNP viremic controllers (LTNP-VC) are long-term non-progressors harboring less than 2000 HIV RNA copies/ml without therapy in 75% of the measurements (n = 63), viremic non-controllers (LTNP-NC) are long-term non-progressors with a viral load between 2000 and 10,000 copies/ml (n = 66). Rapid progressors (RP) are non-LTNP patients with CD4 decline of more than 100 cells/µl per year (n = 35) and normal progressors (NP) are non-LTNP with a CD4 decline less than 100 cells/µl per year (n = 113). Ethical approval was obtained from Ethics Committee of Ghent University Hospital (Reg nr B67020071646) and Instituto de Salud Carlos III (Ref CEI PI 33_2010-v3). All participants provided written informed consent.

**Table 1:** Patient characteristics.

		Ghent cohort (N = 187)	RIS Cohort (N = 138)	Overall (N = 325)	African (N = 34)	Caucasian (N = 291)
	**Age at diagnosis, median years (IQR)**	34 (28–40)	25 (22–30)	29 (24–37)	33 (26–39)	31 (24–36)
	**Follow-up time, median years (IQR)**	8 (6–11)	22 (20–25)	15 (7–22)	8 (6–11)	15 (8–23)
**Sex, N (%)**	**Female**	45 (24,1)	48 (34,5)	93 (28,4)	23 (67,7)	70 (24,1)
	**Male**	142 (75,9)	90 (65,5)	232 (71,6)	11 (32,3)	221 (75,9)
**Ethnicity, N (%)**	**African**	34 (18,2)	0 (0)	34 (10,5)		
	**Caucasian**	153 (81,2)	138 (100)	291 (89,5)		
**Disease progression (N, %)**	**LTNP - Elite controller**	6 (3,2)	42 (30,4)	48 (14,8)	2 (5,9)	46 (15,8)
	**LTNP - Viremic controller**	16 (8,6)	47 (34,1)	63 (19,4)	7 (20,6)	56 (19,2)
	**LTNP - Non controller**	17 (9,1)	49 (35,5)	66 (20,3)	4 (11,8)	62 (21,3)
	**Normal progressor**	113 (60,4)	0 (0,0)	113 (34,8)	19 (55,9)	94 (32,3)
	**Rapid progressor**	35 (18,7)	0 (0,0)	35 (10,8)	2 (5,9)	33 (11,3)
**Current ART, N (%)**	**Yes**	87 (46,4)	18 (12,9)	105 (32,3)	13 (38,2)	92 (31,6)
	**No**	100 (53,6)	120 (87,1)	220 (67,7)	21 (61,8)	199 (68,4)

Overview of the patient characteristics, divided for Ghent and RIS cohort and per ethnicity (Africans, Caucasians). The number of patients per subcategory is presented for sex, ethnicity, disease progression groups and current antiretroviral treatment.

IQR = interquartile range; N = number; LTNP = long-term non-progressor; ART = antiretroviral treatment.

### PSIP1 genotyping

**Figure 1:**
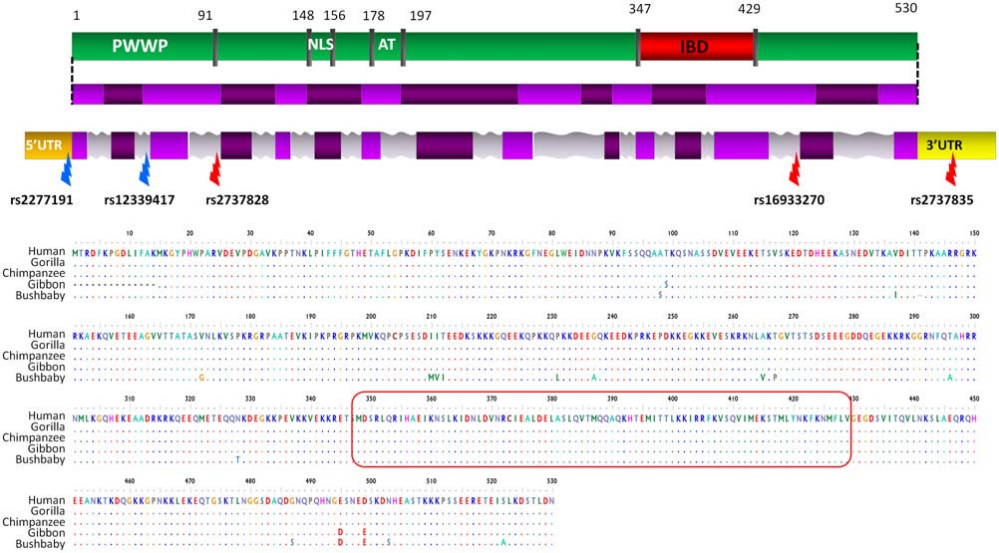
LEDGF/p75 protein domains, gene structure and primate alignment. LEDGF/p75 functional protein domains (green) with the integrase binding domain (IBD, indicated in red) and the PWWP domain, which regulates chromatin association. LEDGF/p75 spliced RNA with different exons (light and dark purple) is linked to the protein domains. The third line represents the *PSIP1* gene region with the coding regions (purple) and non coding regions (introns are grey, 5′UTR and 3′UTR are respectively orange and yellow). The location of the discussed SNPs in this work is indicated (red arrows: SNPs detected after screening; blue arrows: 2 tagSNPs in African cohort). The second panel shows the LEDGF/p75 protein alignment of humans and four primate species based on the reference sequences. The integrase binding domain (red box) is indicated, showing no variation in the four primates. PWWP = proline-tryptophan-tryptophan-proline domain; NLS = nuclear localization signal; AT = adenine-thymine rich DNA binding region; IBD = integrase binding domain; UTR = untranslated region.

After gDNA extraction from whole blood (Blood mini kit, Qiagen), 23 fragments were amplified by PCR, spanning the complete coding region of *PSIP1*, 3′UTR and on average 25 basepairs of the flanking intronic regions (see Figure 1). Two intronic tagSNPs described by Madlala et al. were analyzed as well [37]. Primers were designed with Lightscanner software (Idaho Technologies) and listed in Table S1. Single nucleotide polymorphisms were screened by high resolution melting curve (HRM) analysis (Lightscanner, Idaho Technologies) with high sensitivity detection and auto-grouping [44], [45]. Bi-directional Sanger sequencing with Big Dye Terminator (ABI 3730 xl Sequencer, Applied Biosystems) was performed on all samples with aberrant melting curves for identification of SNPs. This methodology was validated by direct comparison of HRM data with Sanger sequencing in a large group of samples (n = 50). When necessary, detection sensitivity levels were adjusted to assure 100% sensitivity for all variants. All SNPs in the coding region and infrequent SNPs in the non-coding region were re-tested using separate DNA samples to independently verify the results. The impact of SNPs on protein structure was assessed with SIFT (Sorting Intolerant from Tolerant) and Polyphen score (Polymorphism Phenotyping) for variants in the coding region and MaxEnt scan and NNSPlice for intronic variants [46], [47], [48], [49]. In silico evaluation of the amino acid code of *PSIP1* gene product from different primates (Chimpanzee, Gorilla, Gibbon, Bushbaby) was performed based on the consensus sequences found in Ensembl database [50].

### mRNA gene expression analysis

RNA was isolated using Trizol LS reagent (Invitrogen, Carlsbad, USA) from freshly extracted 5×10^6^ peripheral blood mononuclear cells (PBMCs) of patients from the Ghent cohort containing genetic variants that indicated clustering in disease progression groups. Patients not harboring *PSIP1* genetic variants were used as controls. A strict protocol in accordance with MIQE guidelines was followed [51]. After Dnase treatment (Dnase I, Ambion), the RNA integrity was assessed using automated electrophoresis (Experion, Bio-Rad). Reverse transcription was performed on 400 ng RNA with the iScript complementary DNA (cDNA) synthesis kit and random hexamere primers (Bio-Rad). LEDGF/p75 specific mRNA (i.e. not including LEDGF/p52) and HRP2 mRNA expression was quantified by qPCR using dual-labelled hydrolysis probes and LightCycler® 480 Probes Mastermix on a LC480 platform (Roche Diagnostics). After qPCR, the most stable and optimal number of reference genes were validated from a panel of eight reference genes with GeNorm, NormFinder and BestKeeper software [52] (see also Figure S1). The geometric mean of the two most stable reference genes (B2M and YMHAZ) was used as normalization factor for the calculation of relative mRNA expression quantities (qBase Plus, Biogazelle, Ghent, Belgium [53]).

### Statistical analysis

Linkage disequilibrium between different variants was assessed with principal component analysis. Observed minor allele frequencies of individual SNPs were compared to expected frequencies per ethnicity with Fisher exact test (SPSS 19 software). The same analysis was performed to distinguish clustering of alleles in disease progression subgroups. Bonferroni correction for multiple sampling was applied. After normality testing with Shapiro-Wilk, Analysis of variance (ANOVA) was used to determine differences in gene expression between different disease progression groups. Of the genetic variants in the coding region and in the non-coding region with significant clustering in one or more disease progression groups, the influence on gene expression (LEDGF/p75 and HRP2 mRNA) and disease progression (CD4 slope and average viral load) was analyzed for significance by Mann-Whitney U and Kruskal-Wallis test (SPSS 19 software). Pearson correlation and paired T-tests were used to assess correlation between gene expression data and the parameters of disease outcome.

## Results

The study comprised 325 chronically infected HIV-1 patients from a diverse ethnic background with an average follow-up time of 15 years (Table 1). Patients were grouped into five distinct disease progression categories as outlined in Methods. Thirty-four percent of study subjects were either LTNP viremic or elite controllers. CD4 decline or average plasma viral load was independent from HIV subtype. The sensitivity of HRM curve analysis for SNP detection was 100% for all amplicons, the specificity ranged from 82% to 97%. In total 23 individual SNPs were detected, five in coding regions of the gene, six in intronic regions flanking the coding sequences and 12 in the 3′UTR (Table 2 and 3). Most SNPs were previously annotated, two are newly described in this work and submitted for further reference at dbSNP database (NCBI). Of all SNPs, minor allele frequencies (MAF) were calculated and genotype frequencies were determined to be in accordance with Hardy-Weinberg equilibrium. As there can be large differences in MAF between Africans and Europeans, analyses for both ethnicities were separately performed. Two SNPs (rs35678110 and rs13248) were excluded from further analysis, as they did not meet the Hardy-Weinberg law. The observed MAFs of individual SNPs were compared with expected MAFs on population level per ethnicity. In order to detect clustering in disease progression groups, the MAFs in subgroups were calculated and compared. We did not detect linkage disequilibrium between different SNPs with higher MAF.

**Table 2:** Observed genetic variants in the *PSIP1* coding region.

dbSNP rs number	HGVS name	SNP location	African		Caucasian		Amino acid change	SIFT score	POLYPHEN score
	NM_033222.3		MAF exp	MAF obs	MAF exp	MAF obs			
**rs2821529**	c.348T>C	Exon2	0,169	0,107	0,042	0,039	S116S	-	-
**rs139433616**	c.402T>C	Exon5	NA	0,000	NA	0,002	T134T	-	-
**rs188943134**	c.743C>T	Exon8	NA	0,000	NA	0,002	P248L	0,18	0,045
**rs61744944**	c.1415A>T	Exon13	0,034	0,059	NA	0,002	Q472L	0,01	0,007
**rs35678110**	c.1432C>G	Exon14	0,025	0,000	NA	0,004†	L478V	0,21	0,049

† signifies variants not in accordance with Hardy Weinberg law.

Overview of the observed SNPs in the *PSIP1* coding region for LEDGF/p75. Both expected and observed minor allele frequencies (MAF) are shown per ethnicity. The SIFT score ranges from 0 to 1. The amino acid substitution is predicted as damaging if the score is < = 0.05, and tolerated if the score is >0.05. The POLYPHEN score ranges from 0.00 to ≥2.00. The amino acid substitution is predicted possibly damaging if the score is ≥1.50 and probably damaging if the score is ≥2.00.

HGVS = Human Genomic Variation Society; SNP = single nucleotide polymorphism; MAF = minor allele frequency; NA = not assessed; rs = referenced SNP id number; – means no impact due to silent mutation.

**Table 3:** Observed genetic variants in the *PSIP1* non-coding region.

dbSNP rs number	HGVS name	SNP location	African		Caucasian	
	NM_033222.3		MAF exp	MAF obs	MAF exp	MAF obs
**ss536106972**	c.1409-G>C	Intron1	NA	0,000	NA	0,008
**rs2277191**	c.-142+226C>T	Intron1	0,034	0,103	NA	0,011
**rs2795128**	c.73-59G>A	Intron2	0,124	0,109	0,044	0,066
**rs12339417**	c.149+8064G>A	Intron3	0,686	0,638	0,119	0,148
**rs2737828**	c.289-29T>A	Intron4	NA	0,000	0,15	0,028§
**rs16933270**	c.1421-48T>C	Intron14	0,146	0,103	0,000	0,002
**rs188404574**	c.*30T>G	3′UTR	NA	0,000	NA	0,002
**rs144536781**	c.*410G>T	3′UTR	NA	0,016	NA	0,000
**rs114211035**	c.*464A>G	3′UTR	0,085	0,000	NA	0,002
**rs78732111**	c.*592G>C	3′UTR	0,085	0,000	NA	0,002
**rs2737835**	c.*620G>T	3′UTR	0,033	0,129	0,108	0,076
**rs41306098**	c.*684G>A	3′UTR	NA	0,032	0,092	0,056
**rs2665515**	c.*775C>T	3′UTR	0,142	0,094	0,067	0,066
**rs139069294**	c.*790A>G	3′UTR	NA	0,031	NA	0,000
**ss536106976**	c.*815G>T	3′UTR	NA	0,000	NA	0,003
**rs74366322**	c.*1079T>C	3′UTR	0,186	0,200	NA	0,000
**rs13248**	c.*1232G>A	3′UTR	0,611	0,500†	0,004	0,015†
**rs41268959**	c.*1268G>A	3′UTR	NA	0,000	0,025	0,016

† signifies variants not in accordance with Hardy Weinberg law.

§ signifies p<0.001, after Bonferroni correction.

Overview of the observed SNPs in the *PSIP1* non-coding region. Both expected and observed minor allele frequencies (MAF) are shown per ethnicity.

HGVS = Human Genomic Variation Society; SNP = single nucleotide polymorphism; MAF = minor allele frequency; NA = not assessed; rs = referenced SNP id number; ss = submitted SNP id number.

### Genetic variants of PSIP1 in the coding region

**Figure 2:**
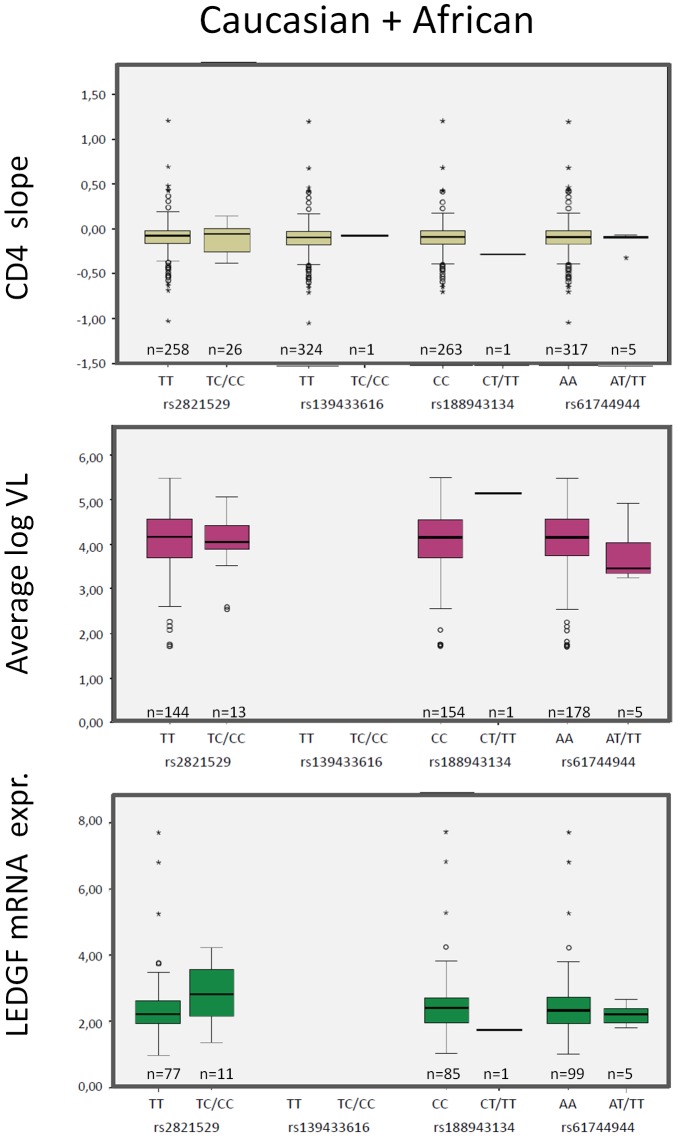
Phenotypic impact of observed genetic variants in the *PSIP1* coding region. Box-plots showing the association of individual observed SNPs in the coding region with CD4 decline (top), average log viral load (middle) and LEDGF/p75 mRNA expression (bottom).The data are combined for Africans and Caucasians. SNPs not in accordance with Hardy-Weinberg law (rs35678110) were excluded. In case of insufficient data to create a boxplot (limited amount of data points) a bar representing the mean of the values is shown.

In the full coding sequence of *PSIP1*, five SNPs were detected, of which two were not altering the encoded amino-acid (silent mutations) and three were non-synonymous SNPs (Table 2). All these variants have been annotated before and had low abundance [54]. Observed minor allele frequencies were compared to expected frequencies per ethnicity, in comparison with data from HapMap and dbSNP database [55], [56]. Fisher exact tests were performed to detect significant lower or higher MAF compared to a randomly selected patient population (Table 2). When comparing the MAFs between the different disease progression groups, no clustering of these variants in one or more subcategories could be detected (data not shown). Kruskal-Wallis test (wild-type versus minor-allele) failed to reveal an association between genetic variants in the coding region and CD4 slope or average viral load. LEDGF/p75 mRNA expression levels did not differ for those patients harboring these SNPs (Figure 2).

Of the two synonymous variants, rs2821529 (S116S) was more abundant and showed comparable MAF to the expected frequencies, both in Africans and Caucasians. No link with disease progression (CD4 decline, average viral load) or LEDGF/p75 mRNA expression could be established (Figure 2). Only one Caucasian patient harbored SNP rs139433616 (T134T), from whom no mRNA for further analysis could be obtained.

The non-synonymous SNPs rs61744944 coding for Q472L missense variant and rs188943134 coding for P248L were both infrequent in Caucasian. SIFT and PolyPhen *in silico* analysis of these variants did not indicate a major impact on protein structure and function [54] (Table 2). The Q472L variant was observed mainly, but not exclusively in Africans and is located outside the known important functional domains. The P248L variant was detected in one Caucasian normal progressor with an average viral load of 5.17 log10 copies/ml. The mutation is also located outside the known important domains.

The non-synonymous SNP (rs35678110), coding for the L478V was present in one Caucasian viremic controller as homozygous allele variant. This amino-acid is situated in a helix-turn-helix motif outside the integrase binding domain. This SNP was however excluded from further analysis due to Hardy-Weinberg disequilibrium. SIFT and Polyphen scores indicated good functional tolerance *in silico.*

### Genetic variants of PSIP1 in intronic regions

In the flanking regions of the coding sequences, four intronic SNPs were detected, of which one previously unknown heterozygous intronic variant in one patient (ss536106972) (Table 3).

**Figure 3:**
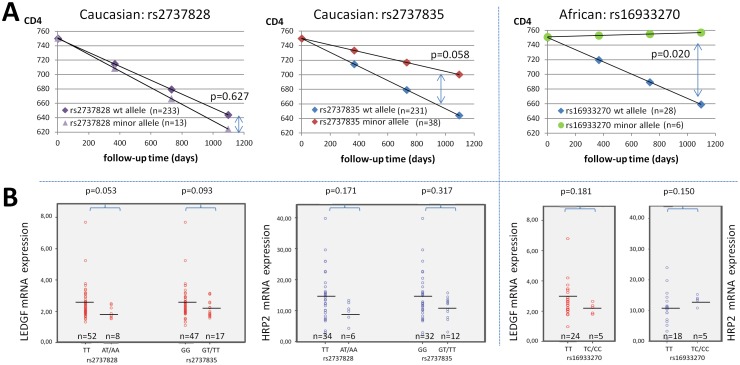
Phenotypic impact of three genetic variants in the *PSIP1* non-coding region. (**A**) Differential CD4 decline for patients carrying wild-type or variant alleles of rs2737828 and rs2737835 (Caucasian) and rs16933270 (African), based on the mean CD4 slope and a similar starting point. P values to estimate significance of difference are indicated. (B) Differential expression of LEDGF/p75 mRNA and HRP2 mRNA in PBMCs from patients carrying wild-type or variant alleles of rs2737828 and rs2737835 (Caucasian) and rs16933270 (African). Horizontal bars indicate the mean expression levels.

Intronic SNP rs2737828, upstream of exon 4, was significantly less abundant in HIV-positive Caucasians compared to Hapmap and dbSNP randomly selected patient population (p<0.0001). There was no significant clustering in patients with long-term non-progression, nor an association with CD4 slope (p = 0.688) or average viral load (p = 0.702) (Figure 3A). A non-significant trend towards lower LEDGF/p75 mRNA expression levels (p = 0.053) was detected in these patients compared to the wild-type genotype. HRP2 expression was not correlated with rs2737828 minor alleles (p = 0.171) (Figure 3B).

For intronic SNP rs16933270, mainly observed in the African subgroup and only in one Caucasian, the MAF was comparable with expected frequencies in Africans, but was relatively more prevalent in the LTNP patients. This variant was associated with slower CD4 decline (p = 0.020) but not with viral load levels, nor with LEDGF/p75 or HRP2 mRNA expression (p = 0.181 and p = 0.150, respectively) (Figure 3A and 3B). A multivariate analysis taking the viral load set-point and LEDGF/p75 mRNA expression into account, confirmed this impact on CD4 slope (p = 0.025). For intronic SNP rs2795128 the observed MAFs were in accordance with the expected frequencies both for Africans as for Caucasians. The minor alleles did not cluster in LTNP groups.

The allele frequencies of two intronic tagSNPs in Africans, i.e. rs2277191 and rs12339417, were additionally determined [37]. Both tagSNPs were abundant in the African subgroup and MAFs were in line with expected frequencies. However, no correlation with CD4 decline or LEDGF/p75 mRNA expression could be confirmed in the cohort. It is interesting to notice that the minor allele of rs12339417 is considered the wild-type allele in Africans.

### Genetic variants in the 3'untranslated region

The 3′ untranslated region (UTR) harbored 12 different genetic variants, one of which was not described previously (Table 3). For rs2737835, a non-significant trend towards association of minor SNP alleles with slower CD4 decline (p = 0.058) was observed in Caucasian but not in African patients (Figure 3A). There was no correlation with average viral load (p = 0.931). The expression levels of both LEDGF/p75 (p = 0.093) and HRP2 (p = 0.317) mRNA were not associated with the presence of these minor alleles (Figure 3B). The SNP rs2737835 is located in a 3′UTR region with higher variability, although no linkage disequilibrium with other variants in this region could be determined.

The other 11 variants did not reveal MAFs aberrant from expected frequencies, eight variants were infrequent both in Africans and in Caucasians. None of these clustered in LTNP subgroups.

### LEDGF/p75 and HRP2 mRNA expression levels

Gene expression analysis was performed on a subset of patients from the Ghent cohort (n = 104). Validation of reference genes for normalization with the GeNorm, NormFinder and BestKeeper software gave congruent results (Figure S1). The geometric mean of the two most stable genes (B2M and YMHAZ) was used for normalization of gene expression data.

**Figure 4:**
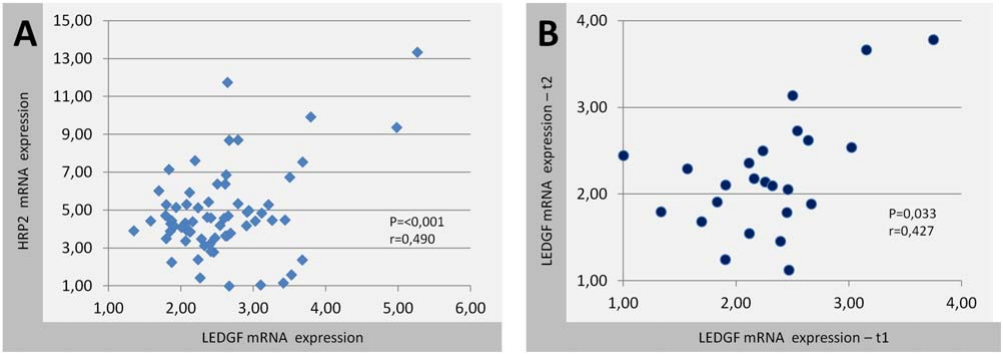
Biological variability and correlation of LEDGF/p75 mRNA with HRP2 mRNA expression. (**A**) Scatter-plot showing LEDGF/p75 mRNA and HRP2 expression in identical patient samples (n = 68). Pearson r-values and p-values are indicated. (B) Scatter-plot showing the biological variation of LEDGF/p75 mRNA expression in two samples from 24 patients at different time points. t1 = time point 1; t2 = time point 2.

We could not demonstrate significant differences in expression levels between patients who received cART and those who were therapy-naïve. No statistical significant differences in either LEDGF/p75 or HRP2 mRNA expression were observed between the five disease progression groups with ANOVA. There was no correlation between LEDGF/p75 expression and the major disease outcome parameters (CD4 decline and average viral load). We could not establish an inverse correlation between LEDGF/p75 and HRP2 expression (Pearson r = 0.490; p = <0.001) (Figure 4A). The biological variability of LEDGF/p75 expression was determined in 24 patients by analyzing samples obtained from two different time-points. Pearson test indicated correlation (r = 0.427; p = 0.033) and the paired T-test could not determine a significant difference in the means between the groups (p = 0.937) (Figure 4B). Inter-patient variability ranged from 7.7-fold expression for LEDGF/p75 till 37-fold for HRP2.

## Discussion

Small molecules inhibiting the interaction between host-factor LEDGF/p75 and the viral enzyme IN form a promising new class of antiretroviral drugs targeting the integration step of HIV-1 replication cycle [28]. Increasing data indicate that genetic variation in host genes can influence HIV-1 disease susceptibility, evolution or therapy response [57]. Since there is little *in vivo* knowledge of the LEDGF/p75-IN interplay, the present study focused on a comprehensive characterization of this host-factor in a large and diverse patient cohort, on the levels of genetic background and mRNA expression.

The data indicate that the coding region of LEDGF/p75 is highly conserved. Of the 35 annotated genetic variants in HapMap (most of them extremely infrequent in a reference population), only five were detected in a cohort of 325 HIV-1 positive patients. Three non-synonymous SNPs were low-abundant and there was no *in silico* indication that they had a major impact on the phenotype. Functional evaluation needs to be performed to confirm these predictions. For rs61744944 (Q472L), experimental data previously showed no alteration of the LEDGF/p75 - IN binding affinity and the near-complete rescue of HIV-1 infection by mutant LEDGF/p75 [37]. However, this does not exclude the possibility of changes in other functions such as integration site distribution. The low MAFs of these variants in the present cohort were insufficient to detect a more subtle impact on disease progression. Patients harboring these variants had normal levels of LEDGF/p75 mRNA expression. One homozygous missense variant (L478V, rs35678110) was detected in one LTNP viremic controller. Unfortunately, no further expression data could be obtained for this patient.

Alignment of consensus sequences of *PSIP1* gene products from different species (Chimpanzee, Gorilla, Gibbons) revealed a highly conserved protein along evolutionary lines, suggesting the important biological function of this gene (Figure 1).

Three SNPs were described with indications for a weak association with HIV disease outcome, situated either in intronic sequences (rs16933270, rs2737828) or in the 3′UTR (rs2737835). In HIV-infected Caucasian patients, rs2737828 was significantly underrepresented and showed a non-significant trend towards lower LEDGF/p75 mRNA expression. This might suggest a protective role of this variant in acquiring HIV-1 without affecting disease evolution, but the cross-sectional study design and the lack of a highly-exposed sero-negative or healthy control arm do not allow to establish susceptibility associations. The underrepresentation of this variant in Caucasian HIV-1 patients needs to be confirmed in different cohorts, since only limited data is available on the geographic distribution of this SNP. In African patients, there was a clustering of rs16933270 minor alleles in patients with slower CD4 decline but no impact on viral load set-point or LEDGF/p75 expression levels. Although similar viral load levels are maintained, some LEDGF/p75 haplotypes could result in a better CD4+ T-cell survival. Low sample size in the African sub-cohort however can introduce bias in these results. The investigation of this variant in a larger cohort of African HIV-1 patients to further assess its impact is recommended. Splice-site finders could not exclude that rs2737828 and rs16933270 had the potential to create a branch point sequence and alter splicing, although no minor splice variants were detected. Variant rs2737835 showed a non-significant tendency towards slower CD4 decline but not towards a lower average viral load. Based on miRNA databases (Patrocles finder), this 3′UTR SNP could be the target for miRNA binding (hsa-mir-1274a), leading to mRNA destabilization and lower protein levels.

The present data did not confirm the association of African tagSNP rs12339417 with delayed CD4 decline in a chronic infection phase. It must be noted that the small sample size of the African sub-cohort and larger genetic diversity in Africans from different regions could explain part of these findings. Besides this, lower LEDGF/p75 expression levels associating with the minor allele were previously only seen in a sero-converter/early infection cohort and it might be possible that LEDGF/p75 levels mainly affect initial HIV infection risk and early replication events. Alternatively, the use of only one reference gene (GAPDH) as normalization strategy in the gene expression assays might have introduced additional bias and hampers comparison [52]. In our assays, normalization with only one reference gene resulted in a 27% increase in variability compared to the standard procedure with two validated reference genes (data not shown).

Relatively stable inter-individual LEDGF/p75 mRNA expression levels were detected in vivo. This variance was not related to genetic polymorphisms in the coding region or 3′UTR. Low levels of LEDGF/p75 expression, linked with decreased integration in transcriptional active regions *in vitro*, did not result in lower viral load [23]. In addition, there was no correlation between low LEDGF/p75 mRNA expression and CD4 decline. Previous in vitro studies revealed that LEDGF/p75 knockdown might be rescued by HRP2, which harbors a similar IN-binding domain [29]. Consequently, the expression of HRP2 was investigated to assess whether low levels of LEDGF/p75 are correlated with higher levels of HRP2 and to see if this rescue mechanism is also present in vivo, but this was not observed. In vitro studies revealed that low levels of LEDGF/p75 are sufficient to completely rescue HIV-1 integration [58]. Other still unknown factors or a more important effect of LEDGF/p75 during early replication might provide further explanation for the observed lack of correlation between LEDGF/p75 expression levels and disease outcome in chronically infected patients. It must be noted that mRNA was extracted from PBMCs, containing not only CD4+ but also CD8+ and B-lymphocytes, and therefore representing average expression levels of these cells. The obtained results did not motivate us to further analyze the 5′ UTR and promoter region of *PSIP1.*

Because of the limited phenotypic effect of non-coding SNPs (on for instance tertiary structure) and the observed conservation of the complete coding region of LEDGF/p75, our data can provide further validation of the LEDGF/p75 – IN interaction as a promising target for antiretroviral treatment. The relatively stable expression of their target shows that LEDGINs could be broadly applicable and provide a good and durable inhibitory effect.

In general, the results of this study underscore the importance of detecting rare variants in genes with a high probability of influencing disease outcome in distinct populations. In contrast with Genome-wide association studies, which are designed to reveal genotype-phenotype relations with common variants, rare variants which show an impact in well-chosen and defined patient populations can help elucidate functional roles of the gene of interest.

## Supporting Information

Table S1 **Overview of primers, cycling conditions and reference sequences.** Overview of primers and cycling conditions for the different PSIP1 gene fragments, including all reference genes used for gene expression analysis. The Ensembl transcript and protein ID of the different PSIP1 gene products from humans and four primates is listed as well.
(DOCX)

Figure S1 **Average expression stability of reference genes.** Average expression stability of the reference genes used in the gene expression assays. Values are calculated with the GeNorm software on at least 15 samples. The gene stability measure M (Y-axis) is the average pairwise variation V of a gene (as indicated in X-axis) with all other genes.
(TIF)

## References

[1] Joint United Nations Programme on HIV/AIDS (2010) Global report: UNAIDS report on the global AIDS epidemic 2010. Accessible at http://www.unaids.org/globalreport/documents/20101123_GlobalReport_full_en.pdf. Accessed 16/09/2012. Geneva.

[2] Antiretroviral Therapy Cohort Collaboration (2008) Life expectancy of individuals on combination antiretroviral therapy in high-income countries: a collaborative analysis of 14 cohort studies. Lancet 372: 293–299. 18657708 10.1016/S0140-6736(08)61113-7 PMC3130543

[3] Arts EJ, Hazuda DJ (2012) HIV-1 Antiretroviral Drug Therapy. Cold Spring Harbor perspectives in medicine 2 4: a007161. 22474613 10.1101/cshperspect.a007161 PMC3312400

[4] Vandekerckhove L, Verhofstede C, Vogelaers D (2009) Maraviroc: perspectives for use in antiretroviral-naive HIV-1-infected patients. The Journal of antimicrobial chemotherapy 63: 1087–1096. 19377064 10.1093/jac/dkp113

[5] Agrawal L, Lu X, Qingwen J, VanHorn-Ali Z, Nicolescu IV, et al (2004) Role for CCR5Delta32 protein in resistance to R5, R5X4, and X4 human immunodeficiency virus type 1 in primary CD4+ cells. J Virol 78: 2277–2287. 14963124 10.1128/JVI.78.5.2277-2287.2004 PMC369216

[6] Friedrich BM, Dziuba N, Li G, Endsley MA, Murray JL, et al (2011) Host factors mediating HIV-1 replication. Virus research 161: 101–114. 21871504 10.1016/j.virusres.2011.08.001

[7] Busschots K, Voet A, De Maeyer M, Rain JC, Emiliani S, et al (2007) Identification of the LEDGF/p75 binding site in HIV-1 integrase. J Mol Biol 365: 1480–1492. 17137594 10.1016/j.jmb.2006.10.094

[8] Cherepanov P (2007) LEDGF/p75 interacts with divergent lentiviral integrases and modulates their enzymatic activity in vitro. Nucleic Acids Res 35: 113–124. 17158150 10.1093/nar/gkl885 PMC1802576

[9] Cherepanov P, Maertens G, Proost P, Devreese B, Van Beeumen J, et al (2003) HIV-1 integrase forms stable tetramers and associates with LEDGF/p75 protein in human cells. J Biol Chem 278: 372–381. 12407101 10.1074/jbc.M209278200

[10] Gijsbers R, Ronen K, Vets S, Malani N, De Rijck J, et al (2010) LEDGF hybrids efficiently retarget lentiviral integration into heterochromatin. Mol Ther 18: 552–560. 20195265 10.1038/mt.2010.36 PMC2839429

[11] Hombrouck A, De Rijck J, Hendrix J, Vandekerckhove L, Voet A, et al (2007) Virus evolution reveals an exclusive role for LEDGF/p75 in chromosomal tethering of HIV. PLoS Pathog 3: e47. 17397262 10.1371/journal.ppat.0030047 PMC1839165

[12] Llano M, Delgado S, Vanegas M, Poeschla EM (2004) Lens epithelium-derived growth factor/p75 prevents proteasomal degradation of HIV-1 integrase. J Biol Chem 279: 55570–55577. 15475359 10.1074/jbc.M408508200

[13] Llano M, Vanegas M, Hutchins N, Thompson D, Delgado S, et al (2006) Identification and characterization of the chromatin-binding domains of the HIV-1 integrase interactor LEDGF/p75. J Mol Biol 360: 760–773. 16793062 10.1016/j.jmb.2006.04.073

[14] Maertens G, Cherepanov P, Pluymers W, Busschots K, De Clercq E, et al (2003) LEDGF/p75 is essential for nuclear and chromosomal targeting of HIV-1 integrase in human cells. J Biol Chem 278: 33528–33539. 12796494 10.1074/jbc.M303594200

[15] Brown-Bryan TA, Leoh LS, Ganapathy V, Pacheco FJ, Mediavilla-Varela M, et al (2008) Alternative splicing and caspase-mediated cleavage generate antagonistic variants of the stress oncoprotein LEDGF/p75. Mol Cancer Res 6: 1293–1307. 18708362 10.1158/1541-7786.MCR-08-0125 PMC2790462

[16] Cohen B, Addadi Y, Sapoznik S, Meir G, Kalchenko V, et al (2009) Transcriptional regulation of vascular endothelial growth factor C by oxidative and thermal stress is mediated by lens epithelium-derived growth factor/p75. Neoplasia 11: 921–933. 19724686 10.1593/neo.09636 PMC2735804

[17] Dietz F, Franken S, Yoshida K, Nakamura H, Kappler J, et al (2002) The family of hepatoma-derived growth factor proteins: characterization of a new member HRP-4 and classification of its subfamilies. Biochem J 366: 491–500. 12006088 10.1042/BJ20011811 PMC1222785

[18] Mediavilla-Varela M, Pacheco FJ, Almaguel F, Perez J, Sahakian E, et al (2009) Docetaxel-induced prostate cancer cell death involves concomitant activation of caspase and lysosomal pathways and is attenuated by LEDGF/p75. Mol Cancer 8: 68. 19715609 10.1186/1476-4598-8-68 PMC2741463

[19] Sharma P, Singh DP, Fatma N, Chylack LT Jr, Shinohara T (2000) Activation of LEDGF gene by thermal-and oxidative-stresses. Biochem Biophys Res Commun 276: 1320–1324. 11027629 10.1006/bbrc.2000.3606

[20] Shinohara T, Singh DP, Fatma N (2002) LEDGF, a survival factor, activates stress-related genes. Prog Retin Eye Res 21: 341–358. 12052388 10.1016/s1350-9462(02)00007-1

[21] Huang TS, Myklebust LM, Kjarland E, Gjertsen BT, Pendino F, et al (2007) LEDGF/p75 has increased expression in blasts from chemotherapy-resistant human acute myelogenic leukemia patients and protects leukemia cells from apoptosis in vitro. Mol Cancer 6: 31. 17451600 10.1186/1476-4598-6-31 PMC1876472

[22] Leoh LS, van Heertum B, De Rijck J, Filippova M, Rios-Colon L, et al (2012) The Stress Oncoprotein LEDGF/p75 Interacts with the Methyl CpG Binding Protein MeCP2 and Influences Its Transcriptional Activity. Molecular cancer research 10: 378–391. 22275515 10.1158/1541-7786.MCR-11-0314 PMC3312617

[23] Ciuffi A, Llano M, Poeschla E, Hoffmann C, Leipzig J, et al (2005) A role for LEDGF/p75 in targeting HIV DNA integration. Nature medicine 11: 1287–1289. 10.1038/nm1329 16311605

[24] De Rijck J, Vandekerckhove L, Gijsbers R, Hombrouck A, Hendrix J, et al (2006) Overexpression of the lens epithelium-derived growth factor/p75 integrase binding domain inhibits human immunodeficiency virus replication. J Virol 80: 11498–11509. 16987986 10.1128/JVI.00801-06 PMC1642583

[25] Emiliani S, Mousnier A, Busschots K, Maroun M, Van Maele B, et al (2005) Integrase mutants defective for interaction with LEDGF/p75 are impaired in chromosome tethering and HIV-1 replication. J Biol Chem 280: 25517–25523. 15855167 10.1074/jbc.M501378200

[26] Engelman A, Cherepanov P (2008) The lentiviral integrase binding protein LEDGF/p75 and HIV-1 replication. PLoS pathogens 4: e1000046. 18369482 10.1371/journal.ppat.1000046 PMC2275779

[27] Vandekerckhove L, Christ F, Van Maele B, De Rijck J, Gijsbers R, et al (2006) Transient and stable knockdown of the integrase cofactor LEDGF/p75 reveals its role in the replication cycle of human immunodeficiency virus. J Virol 80: 1886–1896. 16439544 10.1128/JVI.80.4.1886-1896.2006 PMC1367129

[28] Christ F, Voet A, Marchand A, Nicolet S, Desimmie BA, et al (2010) Rational design of small-molecule inhibitors of the LEDGF/p75-integrase interaction and HIV replication. Nat Chem Biol 6: 442–448. 20473303 10.1038/nchembio.370

[29] Schrijvers R, De Rijck J, Demeulemeester J, Adachi N, Vets S, et al (2012) LEDGF/p75-Independent HIV-1 Replication Demonstrates a Role for HRP-2 and Remains Sensitive to Inhibition by LEDGINs. PLoS Pathog 8: e1002558. 22396646 10.1371/journal.ppat.1002558 PMC3291655

[30] Bushman FD, Malani N, Fernandes J, D'Orso I, Cagney G, et al (2009) Host cell factors in HIV replication: meta-analysis of genome-wide studies. PLoS Pathog 5: e1000437. 19478882 10.1371/journal.ppat.1000437 PMC2682202

[31] Fellay J (2009) Host genetics influences on HIV type-1 disease. Antivir Ther 14: 731–738. 19812435 10.3851/IMP1253 PMC2851194

[32] Fellay J, Ge D, Shianna KV, Colombo S, Ledergerber B, et al (2009) Common genetic variation and the control of HIV-1 in humans. PLoS Genet 5: e1000791. 20041166 10.1371/journal.pgen.1000791 PMC2791220

[33] Fellay J, Shianna KV, Ge D, Colombo S, Ledergerber B, et al (2007) A whole-genome association study of major determinants for host control of HIV-1. Science 317: 944–947. 17641165 10.1126/science.1143767 PMC1991296

[34] Telenti A, Ioannidis JP (2006) Susceptibility to HIV infection–disentangling host genetics and host behavior. J Infect Dis 193: 4–6. 16323124 10.1086/498535

[35] Tozzi V (2010) Pharmacogenetics of antiretrovirals. Antiviral research 85: 190–200. 19744523 10.1016/j.antiviral.2009.09.001

[36] Ballana E, Gonzalo E, Grau E, Iribarren JA, Clotet B, et al (2012) Rare LEDGF/p75 genetic variants in white long-term nonprogressor HIV+ individuals. AIDS 26: 527–528. 22317832 10.1097/QAD.0b013e32834fa194

[37] Madlala P, Gijsbers R, Christ F, Hombrouck A, Werner L, et al (2011) Association of polymorphisms in the LEDGF/p75 gene (PSIP1) with susceptibility to HIV-1 infection and disease progression. AIDS 25: 1711–1719. 21681054 10.1097/QAD.0b013e328349c693 PMC3233670

[38] Garcia-Merino I, de Las Cuevas N, Jimenez JL, Gallego J, Gomez C, et al (2009) The Spanish HIV BioBank: a model of cooperative HIV research. Retrovirology 6: 27. 19272145 10.1186/1742-4690-6-27 PMC2667474

[39] Casado C, Colombo S, Rauch A, Martinez R, Gunthard HF, et al (2010) Host and viral genetic correlates of clinical definitions of HIV-1 disease progression. PLoS One 5: e11079. 20552027 10.1371/journal.pone.0011079 PMC2884031

[40] Grabar S, Selinger-Leneman H, Abgrall S, Pialoux G, Weiss L, et al (2009) Prevalence and comparative characteristics of long-term nonprogressors and HIV controller patients in the French Hospital Database on HIV. AIDS 23: 1163–1169. 19444075 10.1097/QAD.0b013e32832b44c8

[41] Hunt PW (2009) Natural control of HIV-1 replication and long-term nonprogression: overlapping but distinct phenotypes. J Infect Dis 200: 1636–1638. 19852668 10.1086/646610

[42] Okulicz JF, Marconi VC, Landrum ML, Wegner S, Weintrob A, et al (2009) Clinical outcomes of elite controllers, viremic controllers, and long-term nonprogressors in the US Department of Defense HIV natural history study. J Infect Dis 200: 1714–1723. 19852669 10.1086/646609

[43] Pereyra F, Addo MM, Kaufmann DE, Liu Y, Miura T, et al (2008) Genetic and immunologic heterogeneity among persons who control HIV infection in the absence of therapy. J Infect Dis 197: 563–571. 18275276 10.1086/526786

[44] De Leeneer K, Coene I, Poppe B, De Paepe A, Claes K (2008) Rapid and sensitive detection of BRCA1/2 mutations in a diagnostic setting: comparison of two high-resolution melting platforms. Clin Chem 54: 982–989. 18403564 10.1373/clinchem.2007.098764

[45] Wittwer CT, Reed GH, Gundry CN, Vandersteen JG, Pryor RJ (2003) High-resolution genotyping by amplicon melting analysis using LCGreen. Clin Chem 49: 853–860. 12765979 10.1373/49.6.853

[46] Adzhubei IA, Schmidt S, Peshkin L, Ramensky VE, Gerasimova A, et al (2010) A method and server for predicting damaging missense mutations. Nature methods 7: 248–249. 20354512 10.1038/nmeth0410-248 PMC2855889

[47] Kumar P, Henikoff S, Ng PC (2009) Predicting the effects of coding non-synonymous variants on protein function using the SIFT algorithm. Nature protocols 4: 1073–1081. 19561590 10.1038/nprot.2009.86

[48] Eng L, Coutinho G, Nahas S, Yeo G, Tanouye R, et al (2004) Nonclassical splicing mutations in the coding and noncoding regions of the ATM Gene: maximum entropy estimates of splice junction strengths. Human mutation 23: 67–76. 14695534 10.1002/humu.10295

[49] Reese MG, Eeckman FH, Kulp D, Haussler D (1997) Improved splice site detection in Genie. Journal of computational biology 4: 311–323. 9278062 10.1089/cmb.1997.4.311

[50] McLaren W, Pritchard B, Rios D, Chen Y, Flicek P, et al (2010) Deriving the consequences of genomic variants with the Ensembl API and SNP Effect Predictor. Bioinformatics 26: 2069–2070. 20562413 10.1093/bioinformatics/btq330 PMC2916720

[51] Bustin SA, Benes V, Garson JA, Hellemans J, Huggett J, et al (2009) The MIQE guidelines: minimum information for publication of quantitative real-time PCR experiments. Clin Chem 55: 611–622. 19246619 10.1373/clinchem.2008.112797

[52] Vandesompele J, De Preter K, Pattyn F, Poppe B, Van Roy N, et al (2002) Accurate normalization of real-time quantitative RT-PCR data by geometric averaging of multiple internal control genes. Genome Biol 3: RESEARCH0034. 12184808 10.1186/gb-2002-3-7-research0034 PMC126239

[53] Hellemans J, Mortier G, De Paepe A, Speleman F, Vandesompele J (2007) qBase relative quantification framework and software for management and automated analysis of real-time quantitative PCR data. Genome Biol 8: R19. 17291332 10.1186/gb-2007-8-2-r19 PMC1852402

[54] Ensembl Project. Available: http://www.ensembl.org/Homo_sapiens/Transcript/ProtVariations?db=coreg=ENSG00000164985r=9:15464064-15511017t=ENST00000380738 Accessed 06/09/2012.

[55] dbSNP Short Genetic Variations. Availabe: http://www.ncbi.nlm.nih.gov/projects/SNP/ Accessed 06/09/2012.

[56] HapMap project. Available: http://hapmap.ncbi.nlm.nih.gov/cgi-perl/gbrowse/hapmap28_B36/?name=Sequence:NM_033222 Accessed 06/09/2012.

[57] Veloso S, Olona M, Garcia F, Domingo P, Alonso-Villaverde C, et al (2010) Effect of TNF-alpha genetic variants and CCR5 Delta 32 on the vulnerability to HIV-1 infection and disease progression in Caucasian Spaniards. BMC Med Genet 11: 63. 20420684 10.1186/1471-2350-11-63 PMC2877017

[58] Vandegraaff N, Devroe E, Turlure F, Silver PA, Engelman A (2006) Biochemical and genetic analyses of integrase-interacting proteins lens epithelium-derived growth factor (LEDGF)/p75 and hepatoma-derived growth factor related protein 2 (HRP2) in preintegration complex function and HIV-1 replication. Virology 346: 415–426. 16337983 10.1016/j.virol.2005.11.022

