# Supplementary material for: Characterization of LEDGF/p75 Genetic Variants and Association with HIV-1 Disease Progression
Source: PLoS One. 2012 Nov 30;7(11):e50204. doi: 10.1371/journal.pone.0050204 (PMC3511443; doi:10.1371/journal.pone.0050204)
Supplement: Table S1 — Overview of primers, cycling conditions and reference sequences. Overview of primers and cycling conditions for the different PSIP1 gene fragments, including all reference genes used for gene expression analysis. The Ensembl transcript and protein ID of the different PSIP1 gene products from humans and four primates is listed as well. (DOCX) [file pone.0050204.s001.docx]

**Supplementary Table S1: Overview of primers, cycling conditions and reference sequences**

| **Gene name** | **Accession number** |  | **5'-3' PCR primer sequence** |  |  | **Cycling conditions** |
| --- | --- | --- | --- | --- | --- | --- |
| ***PCR*** |  |  |  |  |  |  |
| PSIP1 - Exon 1 | NM_033222.3 | F: | TCGGGGTTCTTCGCTTT | R: | GAGAGGAGGGTAGCACTGC | [95°, 4min], 12cycles [95°, 30s; 60°->48°, 30s; 72°, 40s], 24 cycles [95°, 30s; 48°, 30s; 72°, 40s], [72°, 10min] |
| PSIP1 - Exon 2 | NM_033222.3 | F: | GGATGTTGTGCTCTTGTTTATTC | R: | TAATGTCTACTGCCTAATAACCCT | [95°, 4min], 12cycles [95°, 30s; 62°->50°, 30s; 72°, 40s], 24 cycles [95°, 30s; 50°, 30s; 72°, 40s], [72°, 10min] |
| PSIP1 - Exon 3 | NM_033222.3 | F: | GATAATTGTGTAATAGGGTGTCTTATCTAA | R: | TTTCCTACAGCTAGGATAGTGATTATTC | [95°, 4min], 12cycles [95°, 30s; 62°->50°, 30s; 72°, 40s], 24 cycles [95°, 30s; 50°, 30s; 72°, 40s], [72°, 10min] |
| PSIP1 - Exon 4 | NM_033222.3 | F: | AGAGTGAGACCCTGTCTCTAA | R: | CCAACTCATTTCATTATTTCTTCCTTG | [95°, 4min], 12cycles [95°, 30s; 62°->50°, 30s; 72°, 40s], 24 cycles [95°, 30s; 50°, 30s; 72°, 40s], [72°, 10min] |
| PSIP1 - Exon 5 | NM_033222.3 | F: | GTTTAACAAGGTTTCTTTCATTCCTG | R: | GTATCTACTCTCCCTCAACCC | [95°, 4min], 12cycles [95°, 30s; 62°->50°, 30s; 72°, 40s], 24 cycles [95°, 30s; 50°, 30s; 72°, 40s], [72°, 10min] |
| PSIP1 - Exon 6 | NM_033222.3 | F: | TCGAATTAAACTTTGAGTGCCTACTAT | R: | GCAACACTTTAAATGGACTGGAA | [95°, 4min], 12cycles [95°, 30s; 62°->50°, 30s; 72°, 40s], 24 cycles [95°, 30s; 50°, 30s; 72°, 40s], [72°, 10min] |
| PSIP1 - Exon 7 | NM_033222.3 | F: | AGCAATAATTGTATCTGTAATAAGCTACT | R: | TCGCAGAGATATGTGCTTGTT | [95°, 4min], 12cycles [95°, 30s; 62°->50°, 30s; 72°, 40s], 24 cycles [95°, 30s; 50°, 30s; 72°, 40s], [72°, 10min] |
| PSIP1 - Exon 8 | NM_033222.3 | F: | GAACGTGACAAGTATACATTGTTATG | R: | AACTAAGAATAGAACAGCCAT | [95°, 4min], 12cycles [95°, 30s; 58°->48°, 30s; 72°, 40s], 24 cycles [95°, 30s; 48°, 30s; 72°, 40s], [72°, 10min] |
| PSIP1 - Exon 9 | NM_033222.3 | F: | CCAAGATTATAATTCCATAAGCACTAGTCA | R: | GTCTATTATTTAAAAATTCCATGCTAGCC | [95°, 4min], 12cycles [95°, 30s; 62°->50°, 30s; 72°, 40s], 24 cycles [95°, 30s; 50°, 30s; 72°, 40s], [72°, 10min] |
| PSIP1 - Exon 10 | NM_033222.3 | F: | GGGATTGTGGGCATTTAGTC | R: | AGTTATGTCTATATAACTTCTTTTCCATGT | [95°, 4min], 12cycles [95°, 30s; 62°->50°, 30s; 72°, 40s], 24 cycles [95°, 30s; 50°, 30s; 72°, 40s], [72°, 10min] |
| PSIP1 - Exon 11 | NM_033222.3 | F: | TATACTTAGCCTGTATATAGAAATACTGGT | R: | AAATTTACATACTCATAAGATCATGTGAGA | [95°, 4min], 12cycles [95°, 30s; 62°->50°, 30s; 72°, 40s], 24 cycles [95°, 30s; 50°, 30s; 72°, 40s], [72°, 10min] |
| PSIP1 - Exon 12 | NM_033222.3 | F: | CTTTAGAAACAGATTGTTTAAGTGTTAAG | R: | AACAATATACATTCATCATAATTGTTTTCC | [95°, 4min], 12cycles [95°, 30s; 62°->50°, 30s; 72°, 40s], 24 cycles [95°, 30s; 50°, 30s; 72°, 40s], [72°, 10min] |
| PSIP1 - Exon 13 | NM_033222.3 | F: | ATGTGGATTCTTTGAATTTTGTCATTAC | R: | GCAGTCCTGGCAAATGG | [95°, 4min], 12cycles [95°, 30s; 62°->50°, 30s; 72°, 40s], 24 cycles [95°, 30s; 50°, 30s; 72°, 40s], [72°, 10min] |
| PSIP1 - Exon 14 | NM_033222.3 | F: | TCTGGATCTTTAGTGGACACC | R: | TAACCTTGGAACAGAACTGTGA | [95°, 4min], 12cycles [95°, 30s; 62°->50°, 30s; 72°, 40s], 24 cycles [95°, 30s; 50°, 30s; 72°, 40s], [72°, 10min] |
| PSIP1 - Exon 15 | NM_033222.3 | F: | AATCCTAATTCCAGTTGTACCTTT | R: | TGAAAATATGCTACAACCATGTCTG | [95°, 4min], 32cycles [95°, 30s; 50°, 30s; 72°, 40s], [72°, 10min] |
| PSIP1 - 3'UTR amplicon1 | NM_033222.3 | F: | AGTAAATGTGGGATAAAATCCATTTAGAAA | R: | ACAGAGCACACATTGTTCC | [95°, 4min], 32cycles [95°, 30s; 50°, 30s; 72°, 40s], [72°, 10min] |
| PSIP1 - 3'UTR amplicon2 | NM_033222.3 | F: | ACAGTCTACATTGTGCTACATTATC | R: | TTCCTAAAGACATTTTTAACAACATTCCTC | [95°, 4min], 32cycles [95°, 30s; 50°, 30s; 72°, 40s], [72°, 10min] |
| PSIP1 - 3'UTR amplicon3 | NM_033222.3 | F: | CTCACTAGTAACTAAAACTGAAAACTAAG | R: | TCATATAATTTCAAAACATGAGAAGTATCC | [95°, 4min], 32cycles [95°, 30s; 50°, 30s; 72°, 40s], [72°, 10min] |
| PSIP1 - 3'UTR amplicon4 | NM_033222.3 | F: | AATATTAGGGTCATTTGGCACT | R: | AGAATTCCATCACTTACTCTTGTAAATAG | [95°, 4min], 32cycles [95°, 30s; 55°, 30s; 72°, 40s], [72°, 10min] |
| Intron 1, spanning rs2277191 | NM_033222.3 | F: | TCGCTTCTCGGAATCCCTGCCTT | R: | AAGAGACGCCACCACCTGAAGCA | [95°, 4min], 12cycles [95°, 30s; 62°->50°, 30s; 72°, 40s], 24 cycles [95°, 30s; 50°, 30s; 72°, 40s], [72°, 10min] |
| Intron 2, spanning rs12339417 | NM_033222.3 | F: | TCTGGTCCCTGTTCACCAAACCTGT | R: | AGCCACTAGGAAGCCTGAAGCA | [95°, 4min], 12cycles [95°, 30s; 62°->50°, 30s; 72°, 40s], 24 cycles [95°, 30s; 50°, 30s; 72°, 40s], [72°, 10min] |
| ***qPCR*** | | | | | | |
| LEDGF/p75 cDNA | NM_033222.3 | F: | GAACTTGCTTCACTTCAGGTC | R: | TCGCCGTATTTTTTTCAGTGT | [95°, 1min], 45cycles [95°, 6s; 60°, 6s; 72°, 10s] |
|  |  | probe | TGCAACAAGCTCAGAAACACACAGAGATGA |  |  |  |
| HRP2 cDNA | NM_032631.2 | F: | AAGTTTGCCCTAAAGGTCGACAGC | R: | GTAACGGCGAATCTTCTTCAAGGTGG | [95°, 1min], 45cycles [95°, 3s; 60°, 30s; 72°, 10s] |
| YMHAZ cDNA | NM_003406 | F: | ACTTTTGGTACATTGTGGCTTCAA | R: | CCGCCAGGACAAACCAGTAT | [95°, 1min], 45cycles [95°, 3s; 60°, 30s; 72°, 10s] |
| B2M cDNA | NM_004048 | F: | AGATGAGTATGCCTGCCGTGTGAA | R: | TGCTGCTTACATGTCTCGATCCCA | [95°, 1min], 45cycles [95°, 3s; 60°, 30s; 72°, 10s] |
| PLOD1 cDNA | NM_000302.3 | F: | CAACAACAAGGACAACCGCATCCA | R: | GAATTTGTGCCACTCCCGCTCAAA | [95°, 1min], 45cycles [95°, 3s; 60°, 30s; 72°, 10s] |
| RPL13A cDNA | NM_012423 | F: | CCTGGAGGAGAAGAGGAAAGAGA | R: | TTGAGGACCTCTGTGTATTTGTCAA | [95°, 1min], 45cycles [95°, 3s; 60°, 30s; 72°, 10s] |
| HMBS cDNA | NM_000190 | F: | CCCACGCGAATCACTCTCAT | R: | TGTCTGGTAACGGCAATGCG | [95°, 1min], 45cycles [95°, 3s; 60°, 30s; 72°, 10s] |
| HPRT1 cDNA | NM_000194 | F: | TGACACTGGCAAAACAATGCA | R: | GGTCCTTTTCACCAGCAAGCT | [95°, 1min], 45cycles [95°, 3s; 60°, 30s; 72°, 10s] |
| TBP cDNA | NM_003194 | F: | CAAGCGGTTTGCTGCGGTAATCAT | R: | TGCCAGTCTGGACTGTTCTTCACT | [95°, 1min], 45cycles [95°, 3s; 60°, 30s; 72°, 10s] |
| GAPDH cDNA | NM_002046 | F: | AGCCTCAAGATCATCAGCAATGCC | R: | TGTGGTCATGAGTCCTTCCACGAT | [95°, 1min], 45cycles [95°, 3s; 60°, 30s; 72°, 10s] |
|  | | | | | | |
| **Primate reference sequence** | | **Ensembl Transcript ID** | | **Ensembl Protein ID** | |  |
| Human | |  | [ENST00000380738](http://www.ensembl.org/Homo_sapiens/Transcript/Summary?db=core;g=ENSG00000164985;r=9:15464064-15511017;t=ENST00000380738) |  | [ENSP00000370114](http://www.ensembl.org/Homo_sapiens/Transcript/ProteinSummary?db=core;g=ENSG00000164985;r=9:15464064-15511017;t=ENST00000380738) |  |
| Gorilla | |  | [ENSGGOT00000016215](http://www.ensembl.org/Gorilla_gorilla/Transcript/Summary?db=core;g=ENSGGOG00000016152;r=9:15756712-15804205;t=ENSGGOT00000016215) |  | [ENSGGOP00000015764](http://www.ensembl.org/Gorilla_gorilla/Transcript/ProteinSummary?db=core;g=ENSGGOG00000016152;r=9:15756712-15804205;t=ENSGGOT00000016215) |  |
| Chimpanzee | |  | [ENSPTRT00000038464](http://www.ensembl.org/Pan_troglodytes/Transcript/Sequence_Protein?db=core;g=ENSPTRG00000020786;r=9:15717006-15767195;t=ENSPTRT00000038464) |  | [ENSPTRP00000035550](http://www.ensembl.org/Pan_troglodytes/Transcript/ProteinSummary?db=core;g=ENSPTRG00000020786;r=9:15717006-15767195;t=ENSPTRT00000038464) |  |
| Gibbon | |  | [ENSNLET00000022429](http://www.ensembl.org/Nomascus_leucogenys/Transcript/Summary?db=core;g=ENSNLEG00000017585;r=GL397279.1:26422746-26479436;t=ENSNLET00000022429) |  | [ENSNLEP00000021346](http://www.ensembl.org/Nomascus_leucogenys/Transcript/ProteinSummary?db=core;g=ENSNLEG00000017585;r=GL397279.1:26422746-26479436;t=ENSNLET00000022429) |  |
| Bushbaby | |  | [ENSOGAT00000002182](http://www.ensembl.org/Otolemur_garnettii/Transcript/Summary?db=core;g=ENSOGAG00000002179;r=GL873525.1:12072261-12110961;t=ENSOGAT00000002182) |  | [ENSOGAP00000001950](http://www.ensembl.org/Otolemur_garnettii/Transcript/ProteinSummary?db=core;g=ENSOGAG00000002179;r=GL873525.1:12072261-12110961;t=ENSOGAT00000002182) |  |
