# Supplementary figures and images for: Characterization of LEDGF/p75 Genetic Variants and Association with HIV-1 Disease Progression
Source: PLoS One. 2012 Nov 30;7(11):e50204. doi: 10.1371/journal.pone.0050204 (PMC3511443; doi:10.1371/journal.pone.0050204)

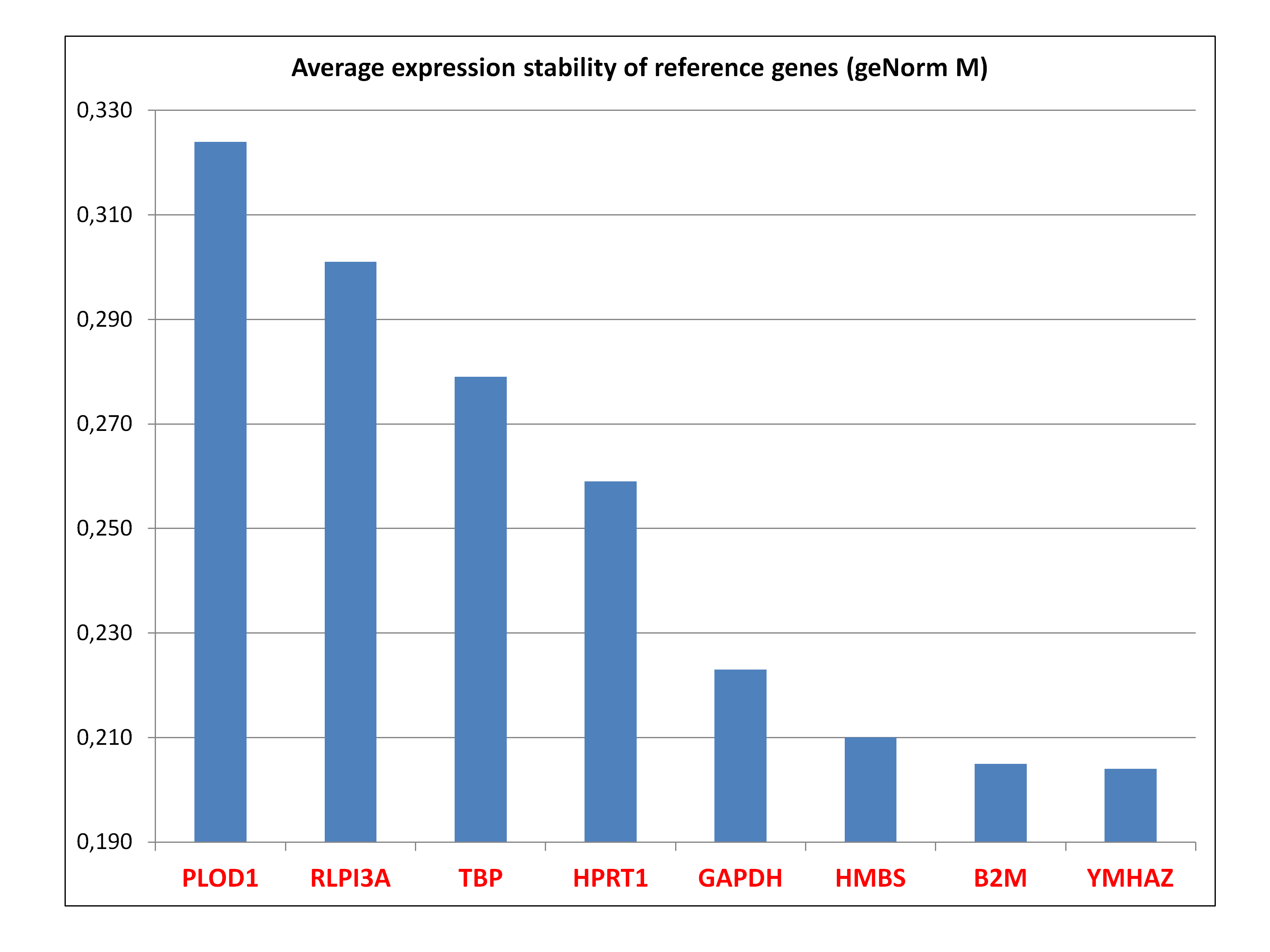

Supplement: Figure S1 — Average expression stability of reference genes. Average expression stability of the reference genes used in the gene expression assays. Values are calculated with the GeNorm software on at least 15 samples. The gene stability measure M (Y-axis) is the average pairwise variation V of a gene (as indicated in X-axis) with all other genes. (TIF) [file pone.0050204.s002.tif]
